# Chronic tobacco smoking, impaired reward-based decision-making, and role of insular cortex: A comparison between early-onset smokers and late-onset smokers

**DOI:** 10.3389/fpsyt.2022.939707

**Published:** 2022-08-26

**Authors:** Aldo Alberto Conti, Alexander Mario Baldacchino

**Affiliations:** ^1^Department of Child & Adolescent Psychiatry, Institute of Psychiatry, Psychology and Neuroscience, King’s College London, London, United Kingdom; ^2^Division of Population and Behavioral Science, University of St Andrews School of Medicine, St Andrews, United Kingdom

**Keywords:** insular cortex, adolescent smokers, cognitive impulsivity, voxel based morphometry, chronic tobacco smoking, neuroimaging, addiction, reward-based decision-making

## Abstract

**Introduction:**

The literature suggests that tobacco smoking may have a neurotoxic effect on the developing adolescent brain. Particularly, it may impair the decision-making process of early-onset smokers (<16 years), by rendering them more prone to impulsive and risky choices toward rewards, and therefore more prone to smoking relapses, in comparison to late-onset smokers (≥16 years). However, no study has ever investigated reward-based decision-making and structural brain differences between early-onset smokers and late-onset smokers.

**Methods:**

Computerized measures of reward-based decision-making [Cambridge Gambling Task (CGT); 5-trials adjusting delay discounting task (ADT-5)] were administered to 11 early-onset smokers (mean age at regular smoking initiation = 13.2 years), 17 late-onset smokers (mean age at regular smoking initiation = 18.0 years), and 24 non-smoker controls. Voxel-based morphometry (VBM) was utilized to investigate the gray matter (GM) and white matter (WM) volume differences in fronto-cortical and striatal brain regions between early-onset smokers, late-onset smokers, and non-smokers.

**Results:**

Early-onset smokers displayed a riskier decision-making behavior in comparison to non-smokers as assessed by the CGT (*p* < 0.01, Cohen’s *f* = 0.48). However, no significant differences (*p* > 0.05) in reward-based decision-making were detected between early-onset smokers and late-onset smokers. VBM results revealed early-onset smokers to present lower GM volume in the bilateral anterior insular cortex (AI) in comparison to late-onset smokers and lower WM volume in the right AI in comparison to late-onset smokers.

**Conclusion:**

Impairments in reward-based decision-making may not be affected by tobacco smoking initiation during early adolescence. Instead, lower GM and WM volume in the AI of early-onset smokers may underline a vulnerability to develop compulsive tobacco seeking and smoking behavior during adulthood.

## Introduction

The World Health Organization (WHO) defines tobacco smoking as the “leading cause of preventable death worldwide.” In fact, it is responsible for the death of approximately 8 million people each year (1). Common chronic and potentially lethal mortal diseases caused by chronic tobacco smoking include cardiopulmonary (e.g., obstructive pulmonary disease) and cerebrovascular (e.g., strokes) conditions, in addition to lung and throat cancers (2).

It is well-known that 90% of adult smokers start regular tobacco use before 21 years of age (3–6). Epidemiological studies revealed a relationship between smoking onset during early adolescence (10–15 years) and the development of higher levels of nicotine dependence compared to smoking onset during subsequent developmental stages of adolescence (7). An international longitudinal study design conducted by Hu et al. (8) revealed a significant negative correlation (*p* < 0.001) between tobacco smoking onset during early adolescence, smoking heaviness, and greater difficulties in quitting smoking during adulthood in 6,684 participants recruited from Australia, the US, and Finland. A longitudinal study conducted on 244 monozygotic twin pairs by Kendler et al. (9) revealed that smokers who started this maladaptive behavior at 14.6 years report higher levels of nicotine dependence and more intense craving for cigarettes compared to twins who started smoking during late adolescence (19.1 years). Another longitudinal study conducted by Paul et al. (10) on 6,559 Australian early adolescents revealed that smoking experimentation at 14–15 years of age increased significantly (*p* < 0.01) the risk of being a chronic smoker 20 years after baseline measurements (men, RR 2.72, 95% confidence interval [CI] 1.74–4.25; women, RR 6.39, 95% CI 2.85–14.33). The risk was higher for early adolescents who experimented with 10 or more cigarettes compared to those who experimented with less than 10 cigarettes (10). While there is consensus about the biopsychosocial factors (e.g., peer pressure, social learning, social deprivation, family members who smoke, family conflict, comorbid psychiatric illness, slow development of frontal brain regions) associated with tobacco smoking initiation during early adolescence [e.g., (5, 11–13)], there is still uncertainty, however, about the relationship between smoking onset during early adolescence and the development of chronic smoking during adulthood (8).

Animal models suggest that the above association may be due to the unique neurostructural and neurochemical alterations caused by nicotine on the developing adolescent brain [for reviews see (14–17)]. Briefly, nicotine exposure during adolescence has been shown to (a) cause an extensive upregulation of nicotinic acetylcholine receptors (nAChRs) and reduce the functioning of metabotropic glutamate type 2 receptors (mGluR2) in the PFC, to (b) influence the expression of genes involved in the neuroplasticity of frontal brain regions, and to (c) cause changes in macromolecular constituents indicative of cell loss (reduced DNA) and altered cell size (protein/DNA ratio) in the cerebral cortex, midbrain, and hippocampus of adolescent rodents (17–19). According to Del Ciampo and Del Ciampo (20), “nicotine use during adolescence has been associated with deleterious effects on development in the prefrontal cortex and hippocampal structure and can lead to irreversible decreased cognitive function, mainly attention, memory and hyperactivity, and severe addiction” (p. 1). Indeed, cognitive impairments such as increased impulsivity and decreased attention have been observed in adolescent rodents after nicotine exposure but not in adult rodents [e.g., (21)]. Heavy metals present in tobacco cigarettes (e.g., lead, arsenic, cadmium) have been also found to disrupt the formation of neural circuits in children and adolescents, and to cause cognitive impairments such as inattention, lower IQ, and learning disabilities (22–24).

In accordance with the above evidence, DeBry and Tiffany (25) proposed the “tobacco- induced neurotoxicity theory of adolescent cognitive development” (TINACD). According to the TINACD, the earlier the initiation of tobacco smoking, the greater the likelihood of suboptimal executive functioning and poor management of impulsivity would be in adult smokers (25). Particularly, deficits in brain areas associated with impulse control and decision-making such as the ventromedial prefrontal cortex (VMPFC), ventrolateral prefrontal cortex (VLPFC), orbitofrontal cortex (OFC), dorsolateral prefrontal cortex (DLPFC), and anterior cingulate cortex (ACC) should cause a poor modulation of reward-driven responses, therefore, prompting smoking relapses during quit attempts (25).

Only a handful of studies have been conducted investigating the association between early smoking initiation during adolescence and neurocognitive impairments during adulthood in chronic tobacco smokers. A study conducted by Mashhoon et al. (7) showed 10 early-onset smokers (mean age at regular smoking onset = 13.2 years) to suffer impairments in response inhibition and attentional performance, as assessed by a GO/No GO task, compared to 10 late-onset smokers (mean age at regular smoking onset = 17.7 years) and 10 non-smokers. A previous study conducted by Jacobsen et al. (26) revealed 42 chronic smokers to suffer impairments in working memory, as assessed by an auditory n-back task, in comparison to 31 non-smokers. A positive correlation was also found between earlier age at smoking initiation (mean age at regular smoking onset = 13.1 years) and performance accuracy on the working memory n-back task. Therefore, indicating “that the performance of subjects who began to smoke at older ages was less impaired than that of subjects who began to smoke at younger ages” [(26), p. 63]. An fMRI study conducted by Galván et al. (27) did not report any response inhibition differences, as assessed by a stop-signal task, between 25 late adolescent smokers (mean age = 19 years) and 25 age-matched non-smoker controls. However, a negative correlation was detected between adolescent smokers’ scores on the Heaviness of Smoking Index (a measure of smoking behavior and dependence during adolescence) and PFC cortical activation during the stop-signal task. Another study (28) reported a correlation between risk-sensitivity, as assessed by the Balloon Analog Risk Task, and neural activation in dorsolateral and ventrolateral prefrontal cortices in 18 adolescent smokers.

Findings from the above studies indicate that regular smoking initiation occurring during the earliest neuro-maturational stages of adolescence may impact negatively cognitive functions, such as working memory, attention, and response inhibition. Furthermore, adolescent smoking behavior may modulate the neural activation in prefrontal brain areas associated with response inhibition and risky decision-making.

Notably, a meta-analysis conducted by Conti et al. (29) revealed that adult chronic smokers suffer mild memory and attentional impairments in comparison to non-smokers. No differences in motor impulsivity/response inhibition were identified between chronic smokers and non-smokers (SMD = 0.105, *p* = 0.24). Moreover, cognitive impulsivity was found to be the most impaired (SMD: 0.881, *p* < 0.005) neurocognitive domain.

Cognitive impulsivity may be defined as “the inability to weigh the consequences of immediate and future events and, consequently, delay gratification” (30). It is characterized by an aberrant reward-based decision-making process, including choice impulsivity (a.k.a delay discounting) and risky decision-making, and has been consistently associated with smoking initiation and maintenance (i.e., relapse) by the literature [e.g., (31, 32)]. However, to the best of our knowledge, no study has ever investigated the possible impact of early smoking initiation on reward-based decision-making impairments (impulsive and risky choices) and their neuroanatomical correlates [gray matter (GM) and white matter (WM) volume] in adult chronic smokers.

Therefore, the aim of the current study was to test the following hypotheses:

1. Early-onset smokers (<16 years) show heightened impulsive and risky choices compared to late-onset smokers (≥16 years) and non-smokers.
2. Early-onset smokers (<16 years) present lower GM and WM volume in brain regions commonly associated with impaired reward-based decision-making (henceforth defined as *a priori* regions of interest) compared to late-onset smokers (≥16 years) and non-smokers. *A priori* regions of interest were determined based on previous studies measuring GM and WM volume differences in chronic smokers compared to non-smokers and on previous studies investigating the relationship between structural brain deficits and impaired reward-based decision-making in chronic smokers [e.g., (33–39)]. Regions of interest included: VLPFC, lOFC, DLPFC, DMPFC, VMPFC, mOFC, ACC, insula, dorsal, and ventral striatum (including adjacent white matter tracts), and anterior corpus callosum.
3. GM and WM volume in *a priori* regions of interest of early-onset smokers (<16 years) is correlated with the heightened risky and impulsive choices manifested by the same early-onset smokers in comparison to late-onset smokers (≥16 years) and non-smokers.

## Materials and methods

### Ethical and other research governance approvals

Ethical approval was granted by the London Bromley Research Ethics Committee (REC) (REC Reference Number: 19/LO/1176) and by the University of St. Andrews Teaching and Research Ethics Committee (UTREC) (UTREC Approval Code: MD14516). Research governance and management approvals were granted by the Tayside National Health System (NHS) R&D department.

### Recruitment

The recruitment procedures are described in our previous study (35). Briefly, chronic smokers and non-smokers have recruited between October 2019 and March 2020 across the south-eastern regions of Scotland through convenience sampling (e.g., internet and newspaper advertisements, word of mouth). Participants needed to attend a screening session (session 1) at the University of St. Andrews School of Medicine and an experimental session (session 2) at Ninewells Hospital, Dundee, on a separate day. During session 1, participants underwent screening procedures and performed computerized measures of reward-based decision-making. During session 2, participants underwent a structural magnetic resonance imaging (MRI) procedure. No more than 3 days were allowed to occur between the two sessions.

All participants provided written informed consent prior to the beginning of the study. They were rewarded £100 for their full participation.

### Inclusion and exclusion criteria

Inclusion and exclusion criteria for participants are shown in Table 1. Objective and subjective screening measures are described in detail in our previous study (35). In summary, the smoking status of participants (non-smoker vs. chronic smoker) was verified through a carbon monoxide (CO) breath test and a salivary cotinine test. Smokers needed to present a CO level ≥ 1 ppm and a salivary cotinine ≥ 2 ng/ml to be included in the study. The presence of illicit substances (e.g., heroin, methamphetamine, cocaine) in the system of the participants was investigated through urine drug analysis. Participants who were found positive for illicit substances were excluded from the study, except occasional cannabis users. In fact, previous research reported a weak association between occasional cannabis smoking and impaired reward-based decision-making, and between occasional cannabis smoking and structural brain abnormalities (40, 41). Additionally, a recent meta-analysis conducted by Lorenzetti et al. (42) did not reveal any GM and WM volume reductions in adolescent cannabis users in comparison to non-cannabis users. In the current study, occasional cannabis use was defined as smoking cannabis two or less times per week. The Mini International Neuropsychiatric Instrument (MINI) version 7.0.2 was utilized to exclude the presence of DSM-V psychiatric disorders (Axis I).

**TABLE 1:** Inclusion and exclusion criteria.

Chronic tobacco smokers	Non-smokers
***Inclusion criteria***	***Inclusion criteria***
Individuals smoking 10 or more cigarettes per day since two or more years	Individuals who never smoked/used tobacco and/or nicotine products
Age range 18–50 years old	Age range 18–50 years old
Ability to understand English and have the capacity to provide informed consent	Ability to understand English and have the capacity to provide informed consent
Currently not enrolled in any smoking cessation program and not taking any pharmacotherapy to aid smoking cessation	
CO ≥ 10 ppm	CO ≤ 4 ppm
Positive to salivary Cotinine (≥20 ng/ml)	Negative to salivary Cotinine (<20 ng/ml)
***Exclusion criteria***	***Exclusion criteria***
Pregnancy	Pregnancy
A score < 3 on the FTND	Individuals consuming nicotine through alternative products of nicotine administration (e.g., vaping), and/or smokeless tobacco (e.g., snuff)
	Ex-smokers
Individuals with current and/or past licit and/or illicit polysubstance use and dependence*	Individuals with current or past licit and/or illicit polysubstance use and dependence
Individuals consuming more than 14 units of alcohol per week	Individuals consuming more than 14 units of alcohol per week
Individuals diagnosed with AXIS 1 psychiatric disorder as defined in DSM-V (except Tobacco Use Disorder)	Individuals diagnosed with AXIS 1 psychiatric disorder as defined in DSM-V
Individuals with a history of serious head injury	Individuals with a history of serious head injury
Individuals affected by chronic communicable and non-communicable conditions (HIV, Diabetes)	Individuals affected by chronic communicable and non-communicable conditions (HIV, Diabetes)
Individuals with metal implants (for MRI purposes)	Individuals with metal implants (for MRI purposes)
Individuals with a neurological disorder (including Dementia)	Individuals with neurological disorder including Dementia
Individuals presenting with DSM-V acute confusional state	Individuals presenting with DSM-V acute confusional state

*Excluding individuals smoking cannabis recreationally mixed with tobacco. CO, Carbon Monoxide; MRI, Magnetic Resonance Imaging; FTND, Fagerström Test for Nicotine Dependence; ppm, parts per million; ng/ml, nanograms per milliliter; DSM-V, Diagnostic statistical manual of mental disorders version 5. Table retrieved from “Neuroanatomical correlates of impulsive choices and risky decision-making in young chronic tobacco smokers: A voxel-based morphometry study” (35).

Chronic smokers were classified into subgroups: early-onset smokers (age at regular smoking onset < 16 years) and late-onset smokers (age at regular smoking onset ≥ 16 years). According to Mashhoon et al. (7), “this age cut-off has been used consistently in substance abuse research as a dividing mark for early- vs. late-onset drug use [e.g., (43–49)]” (p. 48). Age at regular smoking onset was defined as the age at which participants started smoking ≥ 5 tobacco cigarettes per day. Age at regular smoking onset, number of cigarettes smoked daily, units of alcohol consumed per day, and weekly use of cannabis and tobacco were assessed through a screening interview. Smoker participants needed to smoke ≥ 10 cigarettes per day for 2 or more years to be included in the study. Pack-years (a clinical measure of lifetime tobacco smoking exposure) were calculated by the following formula:


Pack⁢Years=N⁢of°⁢cigarettes⁢smoked×day20×N⁢of°⁢smoking⁢years

The severity of nicotine dependence was instead assessed by the Fagerström Test for Nicotine Dependence (FTND) (50). The pre-morbid intelligence quotient (QI) of participants was estimated through the Barona equations (51).

## Instruments

### Reward-based decision-making outcome measures

Computerized measures of reward-based decision-making included the 5-trials adjusting delay discounting task (ADT-5) (52) for impulsive choices and the Cambridge Gambling Task (CGT) (Cambridge Cognition, 2019) for risky choices. A detailed description of these tasks is reported in our previous study (35). Briefly, during the ADT-5, participants needed to choose between £5 available immediately or £10 available at some point in future (e.g., 1 week, 1 month) over five trials. Outcome measures consisted of effective delay 50% (ED50) values computed at the end of the task. As stated by Yoon and Higgins (53), “ED50 represents the delay that is effective in discounting the subjective value of the delayed reinforcer by 50%.” Consistently with the hypothesis proposing early-onset smokers display heightened impulsive choices compared to late-onset smokers and non-smokers, 10£ should have lost 50% of their value at a sooner time point for early-onset smokers compared to both non-smokers and late-onset smokers’ groups.

The CGT measures risky decision-making outside a learning context. It is administered through the Cambridge Neuropsychological Test Automated Battery.^1^ During this task, participants needed to identify a yellow token hidden inside a blue or red box. The ratio of blue and red boxes varied randomly across trials. Participants needed to bet on their decision by selecting a value presented within a circle at the center of an iPad screen. Each participant started with a score of 100 points, and for each correct guess, the value that was betted was added to their total score. On the contrary, the value that was betted was subtracted from their total score for each incorrect guess. Outcome measures for this task consisted of the different facets of the risky decision-making process that are usually impaired in substance users, such as “quality of decision-making,” “risk taking,” “overall proportion bet,” and “risk adjustment” (54–59).

### Neuroimaging

Structural T1-weighted images were acquired through a Siemens 3T Prisma-FIT scanner (Siemens Healthineers, Erlangen, Germany). Images were acquired with a voxel size of 0.8 × 0.8 × 1.0 mm^3^ with whole-brain coverage, repetition time (TR) = 1.9 s, echo time (TE) = 2.64 ms. Flip angle = 9°, FOV = 200 mm, matrix = 256 × 256, 176 slices, slice thickness 1 mm. Scans were reported by a consultant radiologist to rule out the presence of incidental findings.

### Statistical analysis

One-way ANOVAs were utilized to compare early-onset smokers, late-onset smokers, and non-smokers in relation to socio-demographic characteristics (age, social deprivation, pre-morbid IQ, and educational level), patterns of tobacco usage and dependence (cigarettes smoked per day, pack-years, FTND scores, age at regular smoking onset), and daily alcohol usage. Differences between groups in relation to the number of occasional cannabis users and biological sex were instead assessed through chi-squared (χ^2^) tests of associations.

ANCOVAs were conducted to test the null hypothesis of no difference between early-onset smokers, late-onset smokers, and non-smokers in relation to reward-based decision-making outcome measures. These included: ED50 scores of the ADT-5 task; risk taking, overall proportion bet, quality of decision-making, and risk adjustment scores of the CGT. Considering that statistically significant (*p* < 0.05) differences were identified between early-onset smokers and late-onset smokers in relation to biological sex, and between non-smokers, early-onset smokers, and late-onset smokers in relation to educational level and pre-morbid IQ (see Table 2), these socio-demographic variables were inserted as covariates in the ANCOVA analyses.

**TABLE 2:** Socio-demographic characteristics and tobacco smoking characteristics of participants.

	Session 1				Session 2			
	EOS	LOS	NS	Sig^1^.	EOS	LOS	NS	Sig^1^.
**Sociodemographic characteristics**								
*n*	11	17	24		11	12	19	
Age in years (SD)	25.2 (9.3)	30.0 (7.3)	28.5 (9.5)	*p* > 0.05	25.2 (9.3)	31.5 (6.2)	29.7 (9.8)	*p* > 0.05
Sex (%)	36.4% males	82.4% males	54.2% males	EOS > LOS = *p* < 0.05	36.4% males	83.4% males	57.9% males	EOS > LOS = *p* < 0.05
	63.6% females	17.6% females	45.8% females		63.6% females	16.6% females	42.1% females	
Level of education (SD)	3.3 (1.0)	3.6 (0.6)	4.2 (0.6)	NS > EOS = *p* < 0.05	3.3 (1.0)	3.5 (0.6)	4.2 (0.6)	NS > LOS = *p* < 0.05 NS > EOS = *p* < 0.05
Pre-morbid IQ (SD)	101.8 (4.2)	103.5 (3.1)	107.5 (4.1)	NS > EOS = *p* < 0.001 NS > LOS = *p* < 0.01	101.8 (4.2)	103.0 (3.3)	107.9 (4.3)	NS > EOS = *p* < 0.001 NS > LOS = *p* < 0.01
SIMD (SD)	1.8 (1.4)	2.2 (1.6)	2.6 (1.4)	*p* > 0.05	1.8 (1.4)	1.6 (1.3)	2.4 (1.5)	*p* > 0.05
**Tobacco smoking characteristics**				*p* > 0.05				
Cigarettes smoked × day	15.6 (4.7)	14.6 (3.5)	N/A	*p* > 0.05	15.6 (4.7)	15.3 (3.9)	N/A	*p* > 0.05
FTND	5.3 (1.8)	4.8 (1.2)	N/A	*p* > 0.05	5.3 (1.8)	4.9 (1.3)	N/A	*p* > 0.05
Pack years	10.6 (10.1)	10.2 (6.8)	N/A	*p* > 0.05	10.6 (10.1)	11.8 (6.6)	N/A	*p* > 0.05
Age at regular smoking onset in years	13.2 (1.6)	18.0 (2.8)	N/A	LOS > EOS = *p* < 0.001	13.2 (1.61)	18.6 (3.2)	N/A	LOS > EOS = *p* < 0.001
CO level	23.2 (8.4)	21.0 (9.8)	1.2 (0.5)	EOS > NS = *p* < 0.001 LOS > NS = *p* < 0.001	N/A	N/A	N/A	N/A
**Other substances use characteristics**								
Units of alcohol consumed per day (SD)	0.8 (0.8)	0.5 (1.1)	0.2 (0.5)	*p* > 0.05	0.8 (0.8)	0.3 (0.8)	0.2 (0.3)	*p* > 0.05
*n* cannabis smokers	2	3	N/A	*p* > 0.05	2	2	N/A	*p* > 0.05

A mean of 2.8 days separated session 1 from session 2. Data are presented in means and standard deviations (SD) or in percentages (%). Sig^1^ = significance at p < 0.05 two-tailed. Education level scores (1 = left formal education before age 16, 2 = left formal education at age 16, 3 = left formal education at age 18, 4 = undergraduate degree, 5 = master’s degree/post-graduate diploma, 6 = PhD). %, percentage; SIMD, Scottish Index of Multiple Deprivation (1 = most deprived area to 5 = least deprived area); *n*, number of participants; NS, non-smokers; CS, chronic tobacco smokers; CO, carbon monoxide; SD, standard deviation; FTND, Fagerström Test for Nicotine Dependence (0–2 = very low dependence, 2–4 = low dependence, 5 = medium dependence, 6 or more = high dependence); EOS, early-onset smokers; LOS, late-onset smokers; NS, non- smokers.

Assumptions for ANCOVAs included homogeneity of variances, homogeneity of regression slopes, and assumption of normality.

Assumptions of normality and homogeneity of variances were assessed by Shapiro–Wilk’s and Levene’s tests, respectively. Data that violated assumptions of normality and/or of homogeneity variances were log10 transformed. If data still failed assumptions of normality and/or of homogeneity of variances after log10 transformation, non-parametric Kruskal–Wallis H tests were conducted instead of ANCOVAs.

The software SPSS (version 28) was utilized to perform ANOVAs and ANCOVAs analyses. The significance threshold was set at *p* < 0.05. Bonferroni pairwise comparisons were employed to control for Type 1 errors. Effect sizes (Cohen’s *f*) were computed through the software G* Power.^2^

Voxel-based morphometry (VBM) was employed through the software SPM12 to analyze neuroimaging data.^3^ Pre-processing procedures were first conducted by segmenting T1 images into gray and white matter probability maps. Segmented images were subsequently normalized to the Montreal Neurological Institute (MNI) template and modulated to preserve the total amount of GM and WM in each probability map. The last pre-processing procedure consisted in smoothing the modulated images with an 8 mm Gaussian kernel (60). The Computational Anatomy Toolbox^4^ was utilized to extract the total intracranial volume (TIV) for each participant. Brain regions were identified by converting MNI coordinates into Talairach coordinates^5^ and by inserting the converted coordinates into the Talairach Daemon atlas.^6^

Whole-brain two-sample *t*-tests were employed to test for the null hypothesis of no differences between early-onset smokers and late-onset smokers, and between early-onset smokers and non-smokers, in relation to GM and WM volume in *a priori* regions of interest. Therefore, a total of four two-sample *t*-tests were conducted. Whole-brain voxel-wise linear regression models were employed to investigate the associations between outcome measures of reward-based decision-making (CGT and ADT-5 scores) and GM/WM volume in early-onset smokers. Brain-behavior associations were only investigated if early-onset smokers manifested heightened impulsive and/or risky choices in comparison to either late-onset smokers or non-smokers.

Exploratory analyses were also performed by computing whole-brain voxel-wise linear regression models investigating the associations between tobacco exposure variables (FTND scores, pack-years, number of cigarettes smoked per day) and GM/WM volume in early-onset smokers.

*T*-tests and voxel-wise linear regressions were conducted across the whole brain (but limited in scope to *a priori* regions of interest) by utilizing a stringent cluster-extent forming threshold of *p* < 0.01 with a minimum of 100 contiguous voxels per cluster (corrected for multiple comparisons by utilizing Monte-Carlo simulation) (35, 56, 61, 62). Biological sex, TIV, and age were inserted as covariates of no interest in all types of VBM analyses.

## Results

### Socio-demographic, smoking, and other substance use characteristics of the recruited population

**FIGURE 1:**
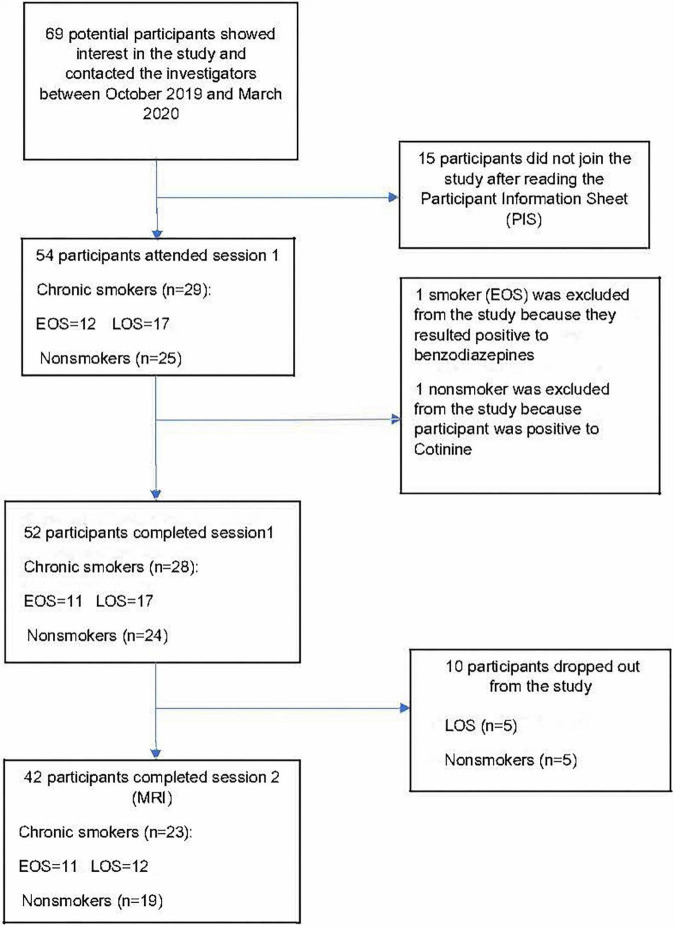
Recruitment flowchart between October 2019 and March 2020. EOS, early onset smokers; LOS, late onset smokers. Figure adapted from “Neuroanatomical correlates of impulsive choices and risky decision making in young chronic tobacco smokers: A voxel-based morphometry study” (35).

The recruitment process is shown in Figure 1. Table 2 shows that the percentage of women participants was significantly (*p* < 0.05) higher in the early-onset smokers’ groups in comparison to both non-smokers and late-onset smokers’ groups. Non-smokers reported a higher level of education (*p* < 0.05) in comparison to early-onset smokers, but not to late-onset smokers (*p* > 0.05). Furthermore, non-smokers reported a higher pre-morbid IQ level compared to both early-onset smokers and late-onset smokers (*p* < 0.05). Moreover, non-smokers did not differ significantly (*p* > 0.05) from early-onset smokers and late-onset smokers in relation to age, Scottish Index Of Multiple Deprivation (SIMD), and units of alcohol consumed per day.

No statistically significant differences were detected between early-onset smokers and late-onset smokers in relation to the number of cigarettes smoked per day, pack-years, and FTND scores (*p* > 0.05). As expected, early-onset smokers differed significantly (*p* < 0.05) from late-onset smokers in relation to “age at regular smoking onset.” Particularly, early-onset smokers started regular smoking at a mean age of 13.2 years, while LOS at 18.0 years. No significant differences were detected between early-onset smokers and late-onset smokers in relation to the number of occasional cannabis smokers (*p* > 0.05). Two occasional cannabis smokers were present in the early-onset smokers’ group, while three occasional cannabis smokers were present in the late-onset smokers’ group. Considering the drop-out of 10 participants (five late-onset smokers, five non-smokers) prior to session 2, sensitivity analyses (ANOVAs) were conducted by just including participants who attended the MRI session. The only difference from socio-demographic comparisons conducted in session 1 consisted in that non-smokers presented a significantly higher (*p* < 0.05) educational level compared to both early-onset smokers and late-onset smokers subgroups. No other differences in results were detected between session 1 and session 2 for socio-demographic and smoking characteristic comparisons (see Table 2).

### Reward-based decision-making outcome measures

ANCOVA analyses revealed significant main effects of group performance on the ADT-5 task (ED50 values), while controlling for biological sex, pre-morbid IQ, and level of education [*F*(2, 46) = 6.06, *p* = 0.005, partial η^2^ = 0.209, Cohen’s *f* = 0.51]. Pairwise comparisons conducted with Bonferroni adjustment revealed statistically significant (*p* = 0.005) higher ED50 values for non-smokers (*M* = 1.86; *SE* = 0.20) compared to late-onset smokers (*M* = 0.74; *SE* = 0.23) with a mean difference of 1.11 (95% CI, 0.29–1.94), but not for non-smokers compared to early-onset smokers (*M* = 0.99; *SE* = 0.28). Furthermore, no statistically significant differences (*p* > 0.05) were detected between early-onset smokers and late-onset smokers in relation to ED50 values.

Regarding risky decision-making, ANCOVA analyses revealed significant main effects of group performance on the CGT for “overall proportion bet” [*F*(2, 46) = 6.07, *p* = 0.005, partial η^2^ = 0.209, Cohen’s *f* = 0.51] and “risky taking” scores [*F*(2, 46) = 5.42, *p* = 0.008, partial η^2^ = 0.191, Cohen’s *f* = 0.48]. Pairwise comparisons conducted with Bonferroni adjustment revealed early-onset smokers (*M* = 0.67; *SE* = 0.05) to display significantly (*p* = 0.003) higher “overall proportion bet” scores in comparison to non-smokers (*M* = 0.42; *SE* = 0.03) with a mean difference of 0.24 (95% CI, 0.07–0.42), but not in comparison to late-onset smokers (*M* = 0.54; *SE* = 0.04). Similarly, early-onset smokers (*M* = 0.71; *SE* = 0.05) displayed significantly (*p* = 0.006) higher “risk-taking” scores in comparison to non-smokers (*M* = 0.47; *SE* = 0.04) with a mean difference of 0.24 (95% CI, 0.05–0.42), but not in comparison to late-onset smokers (*M* = 0.58; *SE* = 0.04). Pairwise comparisons did not reveal any statistically significant difference (*p* > 0.05) between non-smokers and late-onset smokers in relation to both “risk-taking” and “overall proportion bet” scores.

No main effects of group performance on CGT “risk adjustment” scores were detected [*F*(2, 46) = 1.74, *p* = 0.187, partial η^2^ = 0.070, Cohen’s *f* = 0.27]. Furthermore, the Kruskal–Wallis H tests results revealed that early-onset smokers and late-onset smokers did not differ significantly in relation to “quality of decision-making” scores [χ^2^(1) = 0.08, *p* = 0.76]. No statistically significant (*p* > 0.05) differences in relation to “quality of decision-making” scores were also detected between non-smokers and both early-onset smokers and late-onset smokers’ groups. Considering the drop-out of 10 participants (five late-onset smokers and five non-smokers) prior to session 2, sensitivity analyses (ANCOVAs) were conducted to ascertain differences in reward-based decision-making between the three groups of participants at the time of the MRI session. No differences in results were detected between session 1 and session 2. Impulsive choice and risky decision-making scores computed at both sessions are shown in Table 3.

**TABLE 3:** Impulsive choices and risky decision-making scores.

	Session 1				Session 2			
	EOS	LOS	NS	Sig^1^.	EOS	LOS	NS	Sig^1^.
*n*	11	17	24		11	12	19	
**ADT-5**								
ED50*	41.06 (49.71)	16.33 (20.81)	434.45 (922.79)	NS > LOS = *p* < 0.01	41.06 (49.71)	18.90 (23.94)	513.77 (1025.24)	NS > LOS = *p* < 0.05
**CGT**								
Risk taking	0.69 (0.13)	0.59 (0.17)	0.47 (0.18)	EOS > NS = *p* < 0.01	0.69 (0.13)	0.59 (0.20)	0.48 (0.19)	EOS > NS = *p* < 0.05
Overall proportion bet	0.64 (0.14)	0.55 (0.17)	0.43 (0.17)	EOS > NS = *p* < 0.01	0.64 (0.14)	0.55 (0.19)	0.43 (0.18)	EOS > NS = *p* < 0.05
Risk adjustment	0.84 (0.65)	1.85 (1.12)	1.79 (1.30)	*p* > 0.05	0.84 (0.65)	1.82 (1.27)	1.78 (1.39)	*p* > 0.05
Quality of decision making	0.94 (0.08)	0.89 (0.14)	0.93 (0.10)	*p* > 0.05	0.94 (0.08)	0.92 (0.08)	0.93 (0.11)	*p* > 0.05

Data displayed in this table consist of means (M) and standard deviations (SD) of raw ADT-5 and CGT scores. Sig^1^ = significance at *p* < 0.05 two-tailed. EOS, early-onset smokers; LOS, late-onset smokers; NS, non-smokers; CGT, Cambridge Gambling Task; ADT-5, five trials adjusting delay discounting task. *ADT-5 scores (ED50 values) were log10 transformed before conducting the ANCOVAs analyses.

### Neuroimaging: Two-sample *T*-tests

**FIGURE 2:**
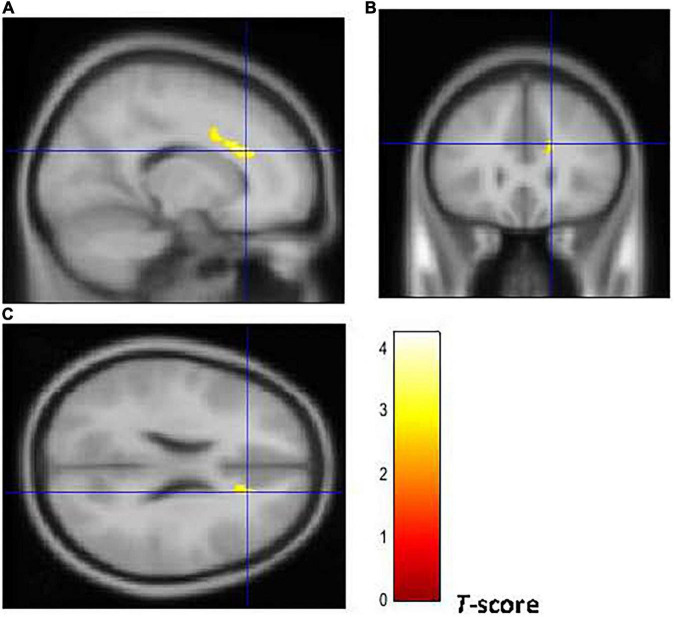
Brain regions of interest displaying lower GM volume in early onset smokers in comparison to non-smokers. The figure shows early onset smokers to display lower GM volume in the right ACC [region of interest centered at 16, 28, 24 MNI coordinates in sagittal **(A)**, coronal **(B)**, and axial **(C)** planes] in comparison to non-smokers. The cluster-forming threshold consisted in *p* < 0.01 with a minimum of 100 voxels per cluster at a whole-brain corrected level. TIV, age, and biological sex were inserted as covariates of no interest.

**FIGURE 3:**
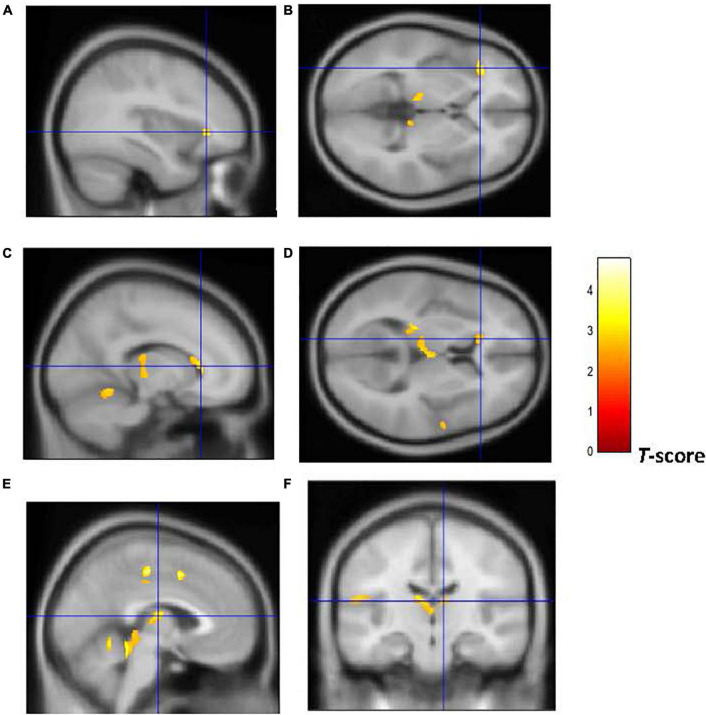
Brain regions of interest displaying lower WM volume in early onset smokers in comparison to non-smokers. The figure shows early onset smokers to display lower WM volume in the left AI [region of interest centered at –36, 30, 0 MNI coordinates in sagittal **(A)** and axial **(B)** planes]; left anterior corpus callosum [region of interest centered at –14, 27, 28 MNI coordinates in sagittal **(C)** and axial **(D)** planes]; and bilateral thalamus [region of interest centered at –3, –14, 12 MNI coordinates in sagittal **(E)** plane, and at 9, –18, 14 MNI coordinates in coronal plane **(F)**] in comparison to non-smokers. The cluster forming threshold consisted in *p* < 0.01 with a minimum of 100 contiguous voxels per cluster at a whole-brain corrected level. TIV, age, and biological sex were inserted as covariates of no interest.

Figures 2, 3 show that early-onset smokers displayed lower GM and WM volume compared to non-smokers in several *a priori* regions of interest. Particularly, early-onset smokers showed lower GM volume in the right ACC (16, 28, 24; *p* = 0.001; *T* = 3.38; *k* = 695; BA 32) and lower WM volume in the left anterior corpus callosum (-14, 27, 28; *p* = 0.003; *T* = 3.07; *k* = 149), bilateral thalamus (left thalamus: –3, 14, 12; *p* = 0.001; *T* = 3.49; *k* = 627) (right thalamus: 9, –18, 14; *p* = 0.004; *T* = 2.90; *k* = 627), and left anterior insula (AI) (-36, 30, 0; *p* = 0.001; *T* = 3.64; *k* = 154; BA 13) in comparison to non-smokers.

**FIGURE 4:**
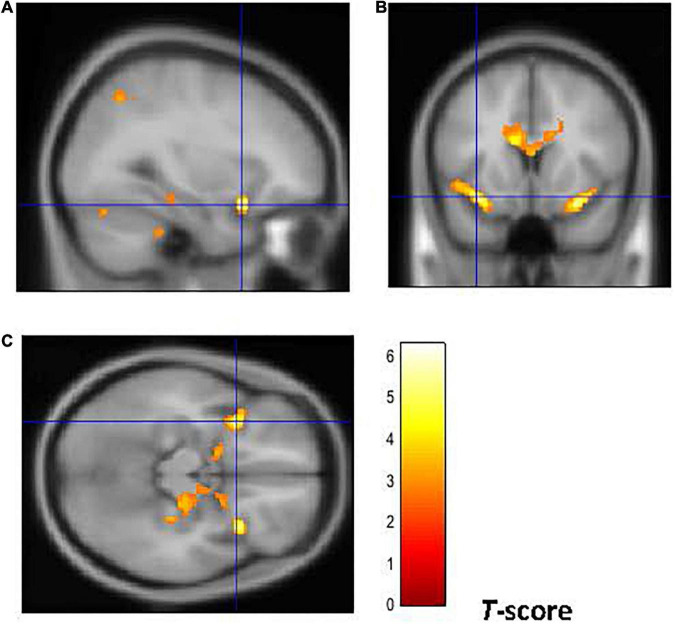
Brain regions of interest displaying lower GM volume in early onset smokers in comparison to late onset smokers. The figure shows early onset smokers to display lower GM volume in the bilateral AI [region of interest centered at 32, 15, –18 MNI coordinates in sagittal plane **(A)**, and at –34, 14, –15 in coronal **(B)** and axial **(C)** planes] in comparison to late onset smokers. The cluster forming threshold consisted in *p* < 0.01 with a minimum of 100 contiguous voxels per cluster at a whole-brain corrected level. TIV, age, and biological sex were inserted as covariates of no interest.

**FIGURE 5:**
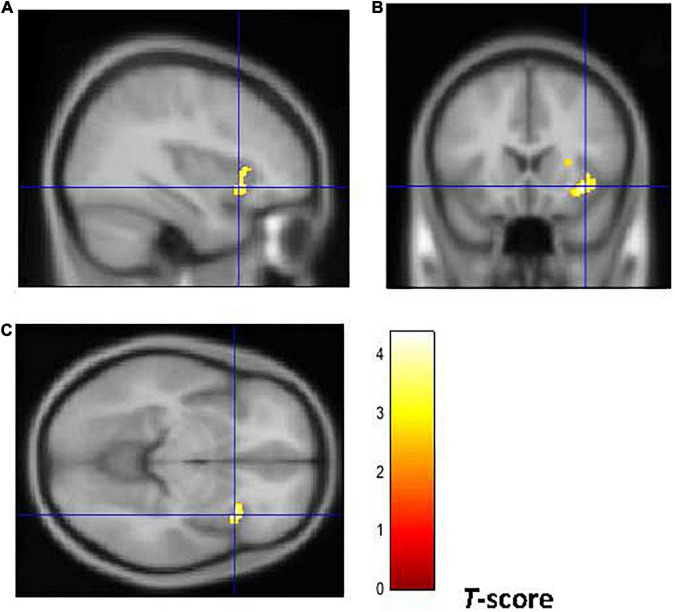
Brain regions of interest displaying lower WM volume in early onset smokers in comparison to late onset smokers. The figure shows early onset smokers to display lower WM volume in the right AI [region of interest centered at 36, 18, –8 MNI coordinates in sagittal **(A)**, coronal **(B)**, and axial **(C)** planes] in comparison to late onset smokers. The cluster forming threshold consisted in *p* < 0.01 with a minimum of 100 contiguous voxels per cluster at a whole-brain corrected level. TIV, age, and biological sex were inserted as covariates of no interest.

Furthermore, as shown in Figures 4, 5, early-onset smokers displayed lower GM volume in the bilateral AI (right AI: 32, 15, –18; *p* = 0.000; *T* = 6.01; *k* = 5,428; BA 13) (left AI: –34, 14, –15; *p* = 0.000; *T* = 5.67; *k* = 948, BA13) and lower WM volume in the right AI (36, 18, –8; *p* = 0.000; *T* = 4.35; *k* = 508, BA13) in comparison to late-onset smokers.

### Neuroimaging: Voxel-wise regression models

Whole-brain voxel-wise regression results revealed negative associations between higher CGT “risk taking” and “overall proportion bet” scores manifested by early-onset smokers in comparison to non-smokers and GM/WM volume in several regions of interest, and these are shown in Table 4 (GM) and Table 5 (WM). Exploratory analyses also showed negative correlations between GM/WM volume in *a priori* brain regions of interest of early-onset smokers and tobacco use characteristics (FTND scores, pack-years, and number of cigarettes smoked per day) (Tables 4, 5).

**TABLE 4:** Whole-brain voxel-wise regression models depicting negative associations between GM volume in early-onset smokers’ brain regions of interest, measures of reward-based decision-making, and tobacco use characteristics.

Covariate of interest	Hemisphere	BA	MNI coordinates (x, y, z)	Peak *T*-values	*P*-values	Cluster size (k)	R^2^	Region of interest
**Cognitive outcome measures**								
CGT overall proportion bet	L	9	–3, 54, 20	8.84	*p* < 0.0001	135	0.906	DMPFC
	L	11	–14, 36, –22	5.35	*p* < 0.005	587	0.800	lOFC
	R	11	32, 46, –16	4.24	*p* < 0.005	163	0.721	lOFC
CGT risk taking	L	9	–3, 54, 21	11.28	*p* < 0.0001	177	0.958	DMPFC
	L	11	–33, 39, –14	4.54	*p* < 0.005	127	0.792	lOFC
**Tobacco use characteristics**								
FTND	L	47	–46, 15, –9	8.00	*p* < 0.0001	160	0.386	VLPFC
	R	9	14, 36, 30	7.86	*p* < 0.0001	227	0.373	DMPFC
	L	32	–8, 38, 9	6.89	*p* < 0.0001	417	0.346	ACC
	R	9	10, 46, 22	5.97	*p* < 0.0001	105	0.352	DLPFC
	R	32	21, 34, 8	5.53	*p* < 0.005	113	0.346	ACC
Pack years	R	N/A	2, 22, 6	7.17	*p* < 0.0001	187	0.093	Caudate
	L	32	–16, 50, –10	5.68	*p* < 0.005	125	0.086	ACC
	L	N/A	–8, –12, –4	5.66	*p* < 0.005	126	0.090	Thalamus
Cigarettes smoked × day	L	N/A	–8, –13, –6	5.42	*p* < 0.005	119	0.506	Thalamus
	L	11	–10, 30, –12	5.09	*p* < 0.005	302	0.489	mOFC

The cluster forming threshold consisted of *p* < 0.01 with a minimum of 100 voxels per cluster. TIV, age, and biological sex were inserted as covariates of no interest. FTND, Fagerström Test for Nicotine Dependence; CGT, Cambridge Gambling Task; BA, Brodmann area; ACC, anterior cingulate cortex; VLPFC, ventrolateral prefrontal cortex; mOFC, medial orbitofrontal cortex; lOFC, lateral orbitofrontal cortex.

**TABLE 5:** Whole-brain voxel-wise regression models depicting negative associations between WM volume in early-onset smokers’ brain regions of interest, measures of reward-based decision-making, and tobacco use characteristics.

Covariate of interest	Hemisphere	BA	MNI coordinates (x,y,z)	Peak *T*-values	*P*-values	Cluster size (k)	R^2^	Region of interest
**Cognitive outcome measures**								
CGT overall proportion bet	R	N/A	22, –4, –12	4.23	*p* < 0.005	104	0.731	Globus pallidus
CGT risk taking	R	N/A	22, –4, –12	4.54	*p* < 0.005	129	0.778	Globus pallidus
**Tobacco use characteristics**								
Pack years	R	N/A	8, 21, –3	7.16	*p* < 0.0001	645	0.908	Caudate
	L	N/A	–9, 12, –9	6.98	*p* < 0.0001	175	0.919	Caudate
Cigarettes smoked × day	R	N/A	6, 20, 0	5.28	*p* < 0.005	217	0.482	Caudate

The cluster forming threshold consisted of *p* < 0.01 with a minimum of 100 voxels per cluster. TIV, age, and biological sex were inserted as covariates of no interest. CGT, Cambridge Gambling Task; BA, Brodmann area.

## Discussion

Overall, the current study did not reveal any significant reward-based decision-making differences between individuals who started regular smoking at approximately 13 years of age in comparison to individuals who started smoking at 18 years of age.

Particularly, early-onset smokers showed specific facets of risky decision-making only compared to NS by betting more points overall CGT trials (overall proportion bet scores) and by betting more points when selecting the more likely outcome (risk-taking scores). No differences were detected between early-onset smokers, non-smokers, and late-onset smokers on the other CGT outcome measures (risk adjustment, quality of decision-making). Furthermore, early-onset smokers did not experience heightened choice impulsivity (ADT-5 scores) compared to both late-onset smokers and non-smokers. Therefore, hypothesis (1) of the current research was not supported. However, early-onset smokers displayed lower GM and WM volume in comparison to non-smokers in several regions of interest, such as ACC, bilateral thalamus, anterior corpus callosum, and AI. Remarkably, early-onset smokers showed lower GM volume in the bilateral AI and lower WM volume in the right AI in comparison to late-onset smokers; thus, supporting hypothesis (2) of the current research.

Negative correlations were detected between the heightened CGT “risk-taking” and “overall proportion bet” scores manifested by early-onset smokers in comparison to non-smokers and GM/WM volume in regions of interest, such as DMPFC, OFC, and white matter tracts adjacent to the globus pallidus. However, considering that CGT “risk-taking” and “overall proportion bet” scores did not differ significantly between early-onset smokers and late-onset smokers, hypothesis (3) of the current study was partially supported.

The above findings show that smoking initiation occurred during the earliest neuro-maturational stages of adolescence (13.2 years) did not impact negatively brain regions associated with impulsive and risky choices. Importantly, no differences in delay discounting rates and risk-taking scores were detected between early-onset smokers and late-onset smokers. Furthermore, early-onset smokers did not show lower GM/WM volume in prefrontal brain areas commonly associated with reward-based decision-making (e.g., VLPFC, ACC) in comparison to late-onset smokers (35, 36).

Counterintuitively, late-onset smokers (but not early-onset smokers) displayed heightened impulsive choices in contrast to non-smokers. These results seem to contradict the TINACD theory of DeBry and Tiffany (25). Indeed, evidence from genetic and twin studies in the addiction medicine literature may support the role of impaired reward-based decision-making/cognitive impulsivity as a pre-morbid trait or neurocognitive endophenotype predisposing individuals toward substance use initiation rather than as a consequence of psychoactive substance use (63–66). For example, a longitudinal-twin study design conducted by Anokhin et al. (65) on 744 adolescent twins (tested at 12 and 14 years) revealed that genetic factors predicted discounting rates at 12 years (30%) and 14 years (51%). Furthermore, discounting rates were found to be predictive of tobacco smoking initiation in the years preceding the analyses (65). Another longitudinal study conducted by Audrain-McGovern et al. (67) on 947 adolescents revealed delay discounting rates measured at baseline (15 years) to be predictive of smoking initiation. However, delay discounting rates did not increase in the years following smoking onset (67). Sparks et al. (68) revealed genetic (0.31%) and non-shared environmental effects (0.67%) to best predict impulsive choices in a sample of 791 adolescents twins aged 17 years. Importantly, “Individuals who chose the immediate reward had over three and a half times the odds of receiving a diagnosis of drug dependence and almost five times the odds of receiving a diagnosis of nicotine dependence” [(68), p. 107]. Euser et al. (69) revealed adolescents with parents suffering from substance use disorder (SUD) to make more risky choices (as assessed by the BART) in comparison to adolescents with parents not suffering from SUD. Other neuroimaging studies identified structural abnormalities in prefrontal brain areas associated with impulsive and risky choices (e.g., ACC, PFC) of substance-dependent individuals and their non-dependent biological siblings [e.g., (70, 71)].

Despite the above evidence, the postulation of impaired reward-based decision-making as a putative pre-morbid neurocognitive endophenotype/vulnerability trait for the tobacco smokers enrolled in the current study remains intuitive due to the cross-sectional nature of the current research and to the absence of genetic and/or neuroimaging data from non-smoker siblings. The most prominent findings from the current study consist in the lower GM volume identified in the bilateral AI of early-onset smokers, and in the lower WM volume identified in the right AI of early-onset smokers, in comparison to late-onset smokers. The fact that early-onset smokers and late-onset smokers were well matched in relation to tobacco exposure and nicotine dependence variables may suggest that these structural brain abnormalities were more impacted by the age of regular smoking onset rather than by chronic tobacco smoking (e.g., early-onset smokers did not report longer pack-years compared to late-onset smokers). Furthermore, no correlations were identified between tobacco exposure variables and GM/WM volume in the AI as revealed by voxel-wise regression results (Tables 4, 5).

The role of the insular cortex in addictions received substantial attention in the last decade [e.g., (72–75)]. Notably, neurofunctional and neurostructural disruptions of the insular cortex have been found to facilitate the transition from an impulsive drug intake to a compulsive drug seeking and taking behavior. This compulsive behavior is a common feature of addictions and substance use disorders (SUDs). According to Lüscher et al. (76), the symptoms listed in DSM-V for SUD reflect a compulsive behavior which is characterized by an excessive time spent searching for the drug of abuse when it is not available, by the prioritization of the search for the drug of abuse over other activities (e.g., familial, social, work-related), by a failure to avoid self-harm, and by the craving for the substance of abuse. Belin-Rauscent et al. (74), who defined the insula as “the neurobiological gate for the development of compulsive behavior,” revealed lower cortical thinness in the bilateral insula of 140 rats to be causally related to heightened motor disinhibition and compulsivity as assessed by a five-choice serial reaction time task (5-CSRTT) and by schedule-induced polydipsia (SIP) task (74). Regarding studies conducted on humans, an MRI study carried out by Grodin et al. (77) showed GM volume and thickness in the bilateral anterior insular cortex of 60 alcohol-dependent individuals (55% of alcohol-dependent individuals were also tobacco smokers) to be negatively correlated with high scores on the “Obsessive–Compulsive Drinking Scale” (OCDS). Morales et al. (78) revealed GM volume in the ventral anterior insular cortex of young cigarette smokers (mean age = 19 years) to be negatively correlated with scores on the “cigarette dependence scale” (CDS) (78). As stated by the authors, “The CDS assesses an individual’s subjective experience of symptoms, such as craving, compulsion to use, levels of stress when unable to smoke, and difficulty quitting or controlling intake” [(78), p. 1,820]. A recent activation likelihood estimation (ALE) meta-analysis conducted by Klugah-Brown et al. (79) on 144 fMRI studies revealed individuals affected by SUDs (cocaine, cannabis, alcohol, and tobacco) and obsessive–compulsive disorder (OCD) share neurofunctional alterations in the bilateral anterior insular cortex. Neurofunctional alterations in the anterior insular cortex have been also associated with impaired processing and regulation of negative moods and emotions in individuals affected by major depressive disorder (80, 81).

The importance of the insular cortex in mediating addiction to tobacco and compulsive cigarette smoking has been also emphasized by brain lesion studies. Remarkably, Naqvi et al. (82) revealed that smoking behavior was disrupted in 19 neurological patients who suffered traumatic insula damage (either left or right) but not in 50 neurological patients who suffered traumatic damage in other brain regions. Similarly, Suñer-Soler et al. (83) revealed that chronic smokers who suffered an acute stroke lesion in the insular cortex were likely to quit smoking within 1 year after brain damage. Gaznick et al. (84) also investigated prospectively (1, 3, 6, and 12 months) the effect of basal ganglia and insula damage on changes in smoking behavior and nicotine dependence in a sample of 63 neurological patients. Their results showed that patients with damages in both basal ganglia and insular cortex presented a more prominent disruption in smoking behavior and nicotine dependence in comparison to patients suffering from damages in the basal ganglia only.

The mechanistic implications of compulsive cigarette seeking and smoking behavior modulated by neurofunctional and neurostructural alterations in the insular cortex are outside the scope of the current study. However, it has been suggested that it may be related to a dysfunctional interoceptive system [see (85) and (75)]. Briefly, a dysfunctional interoceptive system may alter the perception of cue-associated conditioned responses, ultimately leading to craving for cigarettes. Indeed, several studies reported an overactivation of the insular cortex, while participants were exposed to smoking cues during acute tobacco abstinence (86–88). A recent fMRI study conducted by Ghahremani et al. (89) also reported a positive correlation between resting-state functional connectivity of the right ventral AI, craving for cigarettes, and physical symptoms of tobacco withdrawal experienced by smokers during acute abstinence (12 h). Furthermore, findings from a structural MRI study conducted by Perez Diaz et al. (90) showed a negative association between cortical thickness in the right AI and craving symptoms experienced by women smokers after overnight smoking abstinence.

Taken together, results from the current study may suggest that smoking initiation occurring during early adolescence may not be associated with impaired reward-based decision-making. Rather, lower GM and WM volume identified in the AI of early-onset smokers (compared to late-onset smokers) may indicate a vulnerability to develop a compulsive tobacco smoking behavior. This assumption is highly speculative and needs to be investigated further by neuropsychological studies employing neuroimaging techniques (e.g., VBM) and compulsivity tasks/measures.

### Strengths and limitations

The current study presents several strengths. First, the findings of the current study are novel. This is the first study in the literature (to the best of our knowledge) that investigated reward-based decision-making differences and structural brain abnormalities in early-onset smokers in comparison to late-onset smokers by utilizing both cognitive and neuroimaging methods.

Second, the inclusion and exclusion criteria for the current study are stringent, and objective measures (e.g., exhaled CO, salivary cotinine, urine analysis) were employed to assess the smoking status of participants and to identify the presence of illicit substances in their system. Third, early-onset smokers and late-onset smokers were well matched in relation to tobacco exposure variables. The number of occasional cannabis smokers in all participants’ groups was also small.

Limitations of this study include the small sample size. Additionally, causality cannot be directly inferred from the current study results. Due to the cross-sectional design of the current research, it is not possible to infer if the lower GM and WM volume identified in the AI of early-onset smokers is a pre-morbid characteristic or occurred post smoking onset as a result of the neurotoxic effect of tobacco on the adolescent brain. Similarly, it is not possible to infer if impaired reward-based decision-making is a neurocognitive endophenotype/vulnerability trait for the smokers enrolled in the current study. A longitudinal study employing neuroimaging, cognitive, and genetic analyses would be needed to elucidate the causal relationship between early-onset smoking, impairments in reward-based decision-making, and compulsive tobacco seeking and smoking.

## Conclusion

Early-onset smokers made more risky choices in comparison to non-smokers (as assessed by the CGT). Late-onset smokers are discounted at higher rates hypothetical 10£ in comparison to non-smokers (as assessed by the ADT-5). However, no reward-based decision-making differences (impulsive and risky choices) were detected between early-onset smokers and late-onset smokers. VBM analyses revealed early-onset smokers to present lower WM volume in the left AI and bilateral thalamus and left anterior corpus callosum in comparison to non-smokers. Lower GM volume was also detected in the right ACC of early-onset smokers in comparison to non-smokers. Remarkably, early-onset smokers presented lower GM volume in the bilateral AI in comparison to late-onset smokers in addition to lower WM volume in the right AI. Early-onset smokers did not present lower GM/WM volume in other prefrontal brain areas in comparison to late-onset smokers. These findings suggest that smoking onset occurring during the early neuro-maturational stages of adolescence may not be associated with the heightened impulsive and risky choices commonly manifested by chronic tobacco smokers (29, 32, 91, 92). However, lower GM and WM volume in the AI may indicate that early-onset smokers are more vulnerable to developing compulsive tobacco seeking and smoking behavior in comparison to late-onset smokers. This assumption needs to be investigated by future research studies.

## Data availability statement

The raw data supporting the conclusions of this article will be made available by the authors, without undue reservation.

## Ethics statement

The studies involving human participants were reviewed and approved by the London Bromley Research Ethics Committee (REC) (REC Reference Number: 19/LO/1176) and by the University of St. Andrews Teaching and Research Ethics Committee (UTREC) (UTREC Approval Code: MD14516). The patients/participants provided their written informed consent to participate in this study.

## Author contributions

AC and AB were responsible for the study concept and design. AC contributed to the recruitment of participants, acquisition of data, performed the analysis of data, and drafted the manuscript. AB supervised recruitment, screening procedures, assisted with data analysis, interpretation of findings, and provided critical revision of the manuscript for important intellectual content. Both authors critically reviewed the content and approved the final version for publication.

## Footnotes

1
https://www.cambridgecognition.com/cantab/cognitive-tests/executive-function/cambridge-gambling-task-cgt/

2
https://www.psychologie.hhu.de/arbeitsgruppen/allgemeine-psychologie-und-arbeitspsychologie/gpower

3
https://www.fil.ion.ucl.ac.uk/spm/

4
http://www.neuro.uni-jena.de/cat/

5
https://bioimagesuiteweb.github.io/webapp/mni2tal.html

6
http://www.talairach.org/
